# γδ T cells and their clinical application in colon cancer

**DOI:** 10.3389/fimmu.2023.1098847

**Published:** 2023-01-30

**Authors:** Anna Maria Corsale, Marta Di Simone, Elena Lo Presti, Francesco Dieli, Serena Meraviglia

**Affiliations:** ^1^ Central Laboratory of Advanced Diagnosis and Biomedical Research (CLADIBIOR), University of Palermo, Palermo, Italy; ^2^ Department of Health Promotion, Mother and Child Care, Internal Medicine and Medical Specialties (ProMISE), University of Palermo, Palermo, Italy; ^3^ Department of Biomedicine, Neuroscience and Advanced Diagnosis (Bi.N.D.) University of Palermo, Palermo, Italy; ^4^ Institute for Biomedical Research and Innovation (IRIB), National Research Council (CNR)I, Palermo, Italy

**Keywords:** gamma delta T cells, immunotherapy, colon rectal cancer, MHC- unrestricted activation, tumor

## Abstract

In recent years, research has focused on colorectal cancer to implement modern treatment approaches to improve patient survival. In this new era, γδ T cells constitute a new and promising candidate to treat many types of cancer because of their potent killing activity and their ability to recognize tumor antigens independently of HLA molecules. Here, we focus on the roles that γδ T cells play in antitumor immunity, especially in colorectal cancer. Furthermore, we provide an overview of small-scale clinical trials in patients with colorectal cancer employing either *in vivo* activation or adoptive transfer of *ex vivo* expanded γδ T cells and suggest possible combinatorial approaches to treat colon cancer.

## Introduction

Colorectal cancer (CRC) is the third most frequent cancer, with about 1.8 million new cases worldwide in 2018, and the second leading cause of cancer-related deaths worldwide (1). At the onset of CRC, there is a combination of various intrinsic and extrinsic factors, such as genetic factors, gut dysbiosis, dysregulation of the immune system, and environmental factors (2). Although family history is involved in 10%–30% of cases in the pathogenesis of CRC, the majority are sporadic, and the most commonly associated mutations accumulate stepwise, following the so-called adenoma-carcinoma sequence (3). Although the 5-year survival rates for localized tumors are over 90% and 71% for regional tumors, 86% of patients with distant metastases die within 5 years (4). This indicates that, despite the adoption of several screening protocols and currently available standard treatments (surgical resection, radiotherapy, and chemotherapy), there is an urgent need to develop more effective treatments to reduce mortality and tumor relapse.

In this context, immunotherapy appears to be of considerable importance thanks to the unprecedented success obtained in clinical practice. Indeed, in recent years, various studies have been developed on CRC immunotherapy, showing very promising results, especially regarding the application of immune checkpoint inhibitors (ICIs). Pembrolizumab and nivolumab have been authorized for the treatment of advanced and metastatic CRC patients with microsatellite instability-high/DNA mismatch repair deficiency (MSI-H/dMMR) in a chemotherapy-refractory scenario (5). However, ICIs are unsatisfactory in treating microsatellite stable/DNA mismatch repair proficient (MSS/pMMR) CRC because of the low tumor mutation burden and the lack of immune cell infiltration, which are regarded as immune resistance mechanisms (6).. Furthermore, along with chemotherapy, targeted therapies such as monoclonal antibodies targeting epidermal growth factor receptor (EGFR) (e.g., cetuximab) and vascular endothelial growth factor (VEGF) (e.g., bevacizumab) are the gold standard treatments for advanced or metastatic colorectal cancer. At present, all of these treatment options are hampered by the possibility of primary or acquired resistance (7). Therefore, new approaches must be developed to enable more CRC patients to benefit from immunotherapy.

The presence of immune cells in the tumor microenvironment (TME) is known to be a strong predictor of clinical outcome (8). The number of tumor-infiltrating immune cells is referred to as the immunoscore, which is used in conjunction with other characteristics to categorize colorectal cancer. To demonstrate this, Pagès et al. observed a significant link between a 5-year survival rate and a high immunoscore in patients with microsatellite instability or microsatellite-stable CRC; this benefit was not identified when immuscore was low (9). Among the immune cells that make up TME, a noteworthy population is γδ T cells, an innate-like subset of T lymphocytes (10). As well as being located in peripheral blood, they are located in the intestinal epithelium, representing the major population of intraepithelial lymphocytes (IELs). Here, they carry out powerful anticancer and anti-inflammatory actions and are involved in maintaining homeostasis and regulating the microbiome (11, 12). γδ T cells are a valid therapeutic option in the field of anticancer immunotherapy, ensuring efficacy and safety in side effects thanks to their unique recognition capabilities, their remarkable immunosurveillance properties, and their easy handling *in vitro* (13).

This review aims to highlight the role of γδ T cells in colorectal cancer and to summarize the different approaches that allow their use as a therapeutic tool in the treatment of colorectal cancer.

## Human γδ T cells: Characteristics and general functions

γδ T cells are a subset of CD3^+^ lymphocytes that, differently than αβ T cells, express a T-cell receptor (TCR) composed of γ and δ chains. They bridge innate and adaptive immunity, generating quick immune responses with macrophages and neutrophils to several pathogens and helping adaptive immune cells such as T cells and B cells to exert their effector functions. Moreover, thanks to the expression of TCR and typical natural killer (NK) receptors, such as NKG2D and CD94, they can kill target cells and activate other immune cells (14). The vast majority of γδ T cells (~70%) are CD4^−^CD8^−^, ~30% are CD8^+^CD4^−^, and only <1% are CD4^+^CD8^−^ (15). Based on differences in TCR δ-chain usage, human γδ T cells are classified into three cellular subsets with specific tropisms: Vδ1^+^, Vδ2^+^, and Vδ1^−^Vδ2^−^ (16). Vδ1^+^ T cells are predominantly expressed in mucosal and epithelial tissues and represent a large portion of human intestinal IELs (17). They show cytotoxic activities thanks to their ability to recognize stress antigens expressed by transformed cells, such as MHC class I-related molecules A and B (MICA/MICB) and UL16-binding proteins *via* the NKG2D receptor (18) (19). Also, they recognize self-glycolipids presented by CD1c and CD1d (20) (21) and are expanded during *Cytomegalovirus* (CMV) infection (22). The Vδ1 chain can be paired with several Vγ chains (Vγ2, Vγ3, Vγ4, Vγ5, Vγ8, and Vγ10) (23), but in the gut, it is paired with the Vγ4 chain (12).

The most representative γδ T-cell subset expresses the Vδ2 chain typically associated with Vγ9 chain. These cells are generally referred to as Vγ9Vδ2 T cells (24), and they account for 1%–10% of circulating T cells and 50%–95% of the total γδ T-cell compartment (25). The distinctive feature of Vγ9Vδ2 T cells is to recognize and respond to phosphorylated metabolites called phosphoantigens (P-Ags) (14). These are intermediates of the endogenous mevalonate (MVA) pathway (e.g., isopentenyl pyrophosphate (IPP)), upregulated during tumor transformation, or the exogenous 1-deoxy-d-xylulose-5-phosphate (MEP) pathway (e.g., (E)-4-hydroxy-3-methyl-but-2-enyl pyrophosphate (HMBPP)), upregulated during infections (26, 27). As a rule, P-Ags recognition is not MHC-restricted but requires butyrophilin (BTN)2A1 and BTN3A1, two type-I glycoproteins of the B7 family. The BTN2A1–BTN3A1 complex contacts with the γδ TCR in two sites: BTN2A1 binds to the Vγ9 domain, whereas BTN3A1 may bind to the Vδ2 and γ-chain regions on the opposite side of the TCR (28). Once activated, Vγ9Vδ2 T cells kill infected, stressed, and tumor cells using the same mechanisms as NK and CD8^+^ T cells. Moreover, after activation, they interact with B cells, promoting CD4^+^ and CD8^+^ T-cell priming and inducing maturation of dendritic cells (DC) and survival of monocytes and neutrophils (14, 29, 30). These properties let Vγ9Vδ2 T cells orchestrate an innate-like response against pathogens and tumor cells. Recently, Davey et al. have described a population of Vγ9^−^Vδ2^+^ T cells that do not respond to P-Ags and have adaptive-like functions (31).

The Vδ1^−^Vδ2^−^ compartment consists of distinct minor subsets, such as Vδ3^+^ and Vδ5^+^ T cells. Vδ3^+^ T cells amount to 0.2% of T lymphocytes in the blood and are also located in the liver and gut. Currently, its paired γ chain is unknown. Due to a lack of specific antibodies for their identification, generally, they are characterized as Vδ1−Vδ2−cells (32).Vδ3^+^ T cells recognize CD1d molecules expressed on target cells, have cytotoxic activity, and release Th1-, Th2-, and Th17-like proinflammatory cytokines (32). A recent study by Petrasca et al. described a role for this subset in the maturation of B cells and the production of IgM antibodies, without promoting class-switching (33).

Vδ5^+^ γδ T cells are the rarest subset; in particular, Willcox et al. described a clone of these Vδ5^+^Vγ4^+^ T cells that recognize the endothelial protein C receptor expressed by stressed human cells (34).

## Human γδ T cells and their responses to tumors

γδ T cells are one of the components of the tumor immune milieu and are well-known for influencing the antitumor immune response to a broad range of tumors (35). Indeed, γδ T cells are recruited into various types of malignancies, including breast cancer (36), melanoma (37), and lung cancer (38). In particular, with regard to colon cancer, we have previously shown that γδ T lymphocytes, of which Vδ1 was the most represented subset, amounted to 4.5% of the total leukocyte population in CRC (39). In addition, Corvaisier et al. have described a Vγ9Vδ2 TCR clone isolated from colon cancer ascites (40). Recently, a TCR γδ^+^CD103^+^PD-1^+^ subset was exclusively found by high-dimensional single-cell mass cytometry in MMR-deficient CRC, accounting for 8.4% of CD45^+^ cells (41, 42).

Despite some studies that have identified the presence of γδ T cells among tumor-infiltrating lymphocytes (TILs) as a positive prognostic factor, it is not yet fully known whether they can promote or hamper tumor growth (43). Particularly, Gentles et al., analyzing the gene expression profile of 18,000 tumor fragments from 39 types of malignancies on the prognostic role of the individual infiltrating cell populations, showed that the neutrophils and plasma cells present in tumor infiltrate have a significant but opposite role in different tumors, while the presence of γδ T cells was found to be the most favorable prognostic factor in analyzed tumors, both hematological and solid (44), including CRC. However, Tosolini et al., applying the CIBERSORT algorithm, showed that in Gentle’s study, the presence of Vγ9Vδ2 was wrongly identified as CD4^+^CD8^+^ T cells or NK and *vice versa* (45). Therefore, using an improved deconvoluted CIBERSORT matrix to investigate the abundance of TCR Vγ9Vδ2, they found that CRC patients with a high TCR Vγ9Vδ2 infiltration had a better survival rate than those with a low infiltration (45). Similarly, we have shown by transcriptomic analysis of a cohort of 557 CRC patients that 5-year disease-free survival was significantly higher in CRC patients with higher γδ TILs (39). In addition, the decrease of NKp46^+^/Vδ1 IELs in tumor-free specimens from CRC patients correlated with a higher risk of metastasis (12).

In contrast to these findings, a previous study highlighted the role of IL-17A-producing γδ TILs in the promotion of tumor progression in patients with CRC (46), but these results were not confirmed in subsequent studies (39).

This twofold role can be linked to the plasticity of γδ T cells, which includes the capacity to differentiate toward different functional cell subsets based on the microenvironment, which can direct their polarization. This implies that the positive or negative correlation with the tumor prognosis can be attributed to the specific γδ T cell subsets recruited or resident at the tumor site (10).

### Protumor activities of γδ T cells

Notwithstanding that γδ T cells exert antitumor effects, it is not surprising that they also have protumor activity due to their plasticity. Indeed, after recruitment to the tumor site, they can acquire a phenotype that mirrors Tfh, Treg, Th1, Th2, and Th17, influenced by a plethora of cytokines present in the tumor microenvironment (47). Particularly, the polarized γδ T17 and γδ Treg cells, which produce IL-17A or TGF-β, respectively, have been reported to support cancer progression and be associated with poor survival in the gallbladder (48), ovarian (49), and colorectal cancer (39, 46).

In a study by Wu et al., γδ T cells were the major cellular source of IL-17A in human CRC. Activated inflammatory DC polarized Vγ9Vδ2 T cells into IL-17A-producing γδ T cells. Together with the secretion of IL-8, TNF-α, and granulocyte-macrophage colony-stimulating factor (GM-CSF), they induced an immunosuppressive microenvironment in human CRC, thus promoting tumor progression. The tumor infiltration of IL-17A-producing γδ T cells was positively associated with advanced tumor stages of human CRC and with other clinicopathological features, including tumor size, lymphatic and vascular invasion, and lymph node metastasis (46). However, in a subsequent study (39) with two very large cohorts of CRC patients, we failed to confirm the results of Wu and colleagues. In ovarian cancer, γδ T cells and their Vδ1 subset produced IL-17A, impaired antitumor responses, and enhanced immunosuppressive activities, as documented by their ability to inhibit the proliferation of naive CD4^+^ T cells upon co-culture with ovarian cancer tissue supernatants (49). Rong et al. described an imbalance in Vδ1 and Vδ2 subset distribution that may contribute to the growth of rectal cancer. Unlike Vδ2 T cells, Vδ1^+^ TILs were significantly increased, when compared with para-carcinoma tissues, and had strong inhibitory effects (50). Moreover, a CD39^+^ γδ Treg subset was identified in the human CRC, showing a superior immunosuppressive activity than CD4^+^ or CD8^+^ Tregs *via* the adenosine pathway. CD39^+^ γδ Treg cells also established an immunosuppressive milieu due to the chemo-attraction of myeloid-derived suppressor cells (MDSCs) in an IL-17A and GM-CSF-dependent manner. In addition, their infiltration was correlated with unfavorable clinicopathological features in CRC (51). Recently, in the microenvironment of human breast cancer, a regulatory subset of CD73^+^ γδ T cells was identified, which exerted immunosuppressive functions through the production of IL-10, IL-8, and adenosine (52).

Consequently, this evidence, although still very preliminary, suggests that γδ T cells may establish an immunosuppressive microenvironment to inhibit antitumor immune responses, evade immune surveillance, and promote the progression of cancer.

### Antitumor activities of γδ T cells

**Figure 1:**
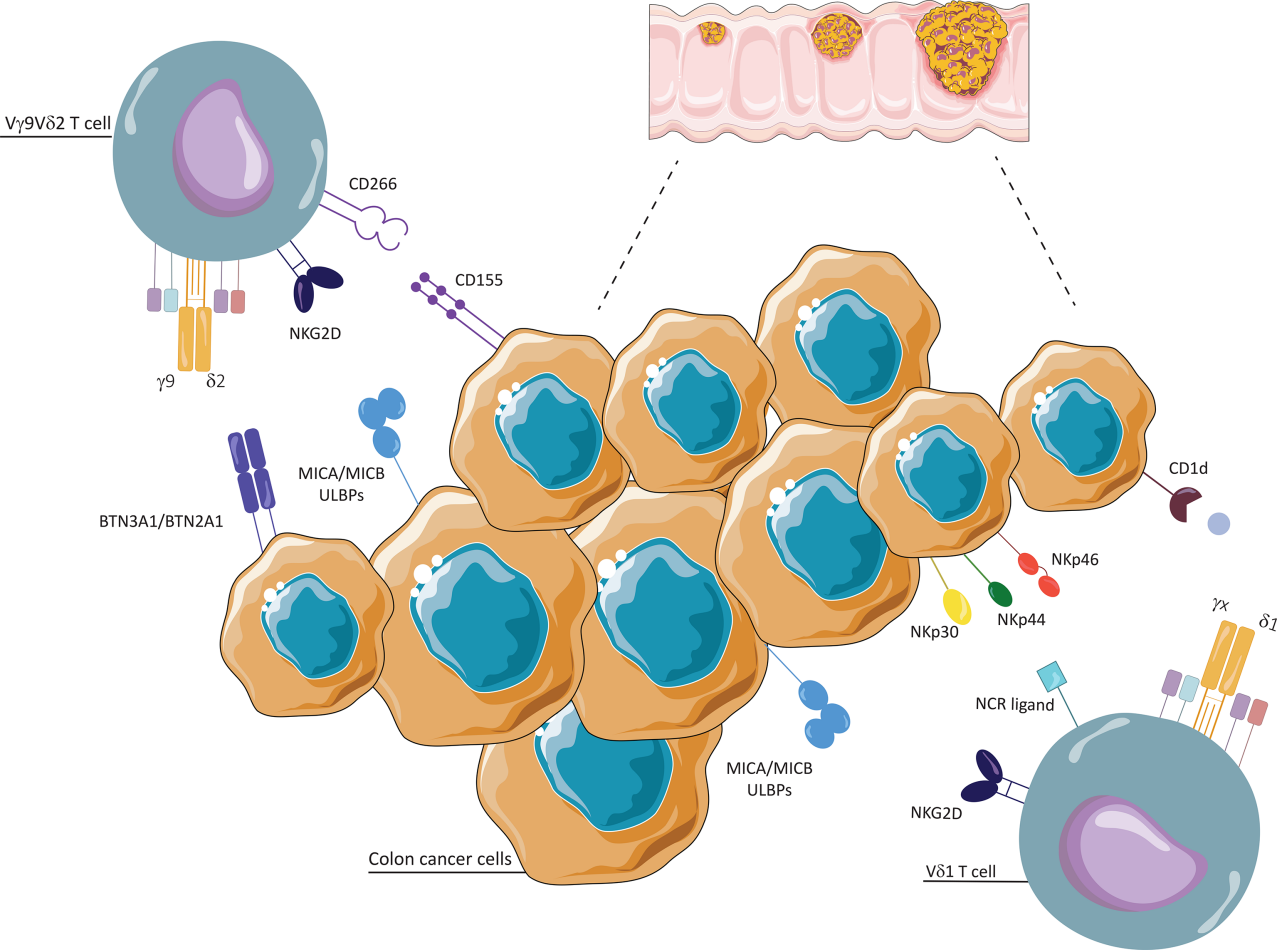
Interaction between γδ T cells and colon cancer cells. Vδ1 and Vδ2 T cells conduct cytotoxic activities thanks to their ability to recognize stress antigens expressed by transformed cells, such as MHC class I-related molecules A and B (MICA/MICB) and UL16-binding proteins (ULBPs), via NKG2D receptor. Vδ1 T cells recognize self-glycolipids presented by CD1c and CD1d, and tumor cells through natural cytotoxicity receptors (NKp30, NKp44, NKp46). Vδ2 T cells recognize and respond to phosphoantigens (P-Ags), through unrestricted MHC manner, with two types I glycoproteins of the B7 family, butyrophilin (BTN)2A1 and BTN3A1. They interact with tumor cells through CD226 (DNAM-1)/CD155 (Nectin-2).

**Figure 2:**
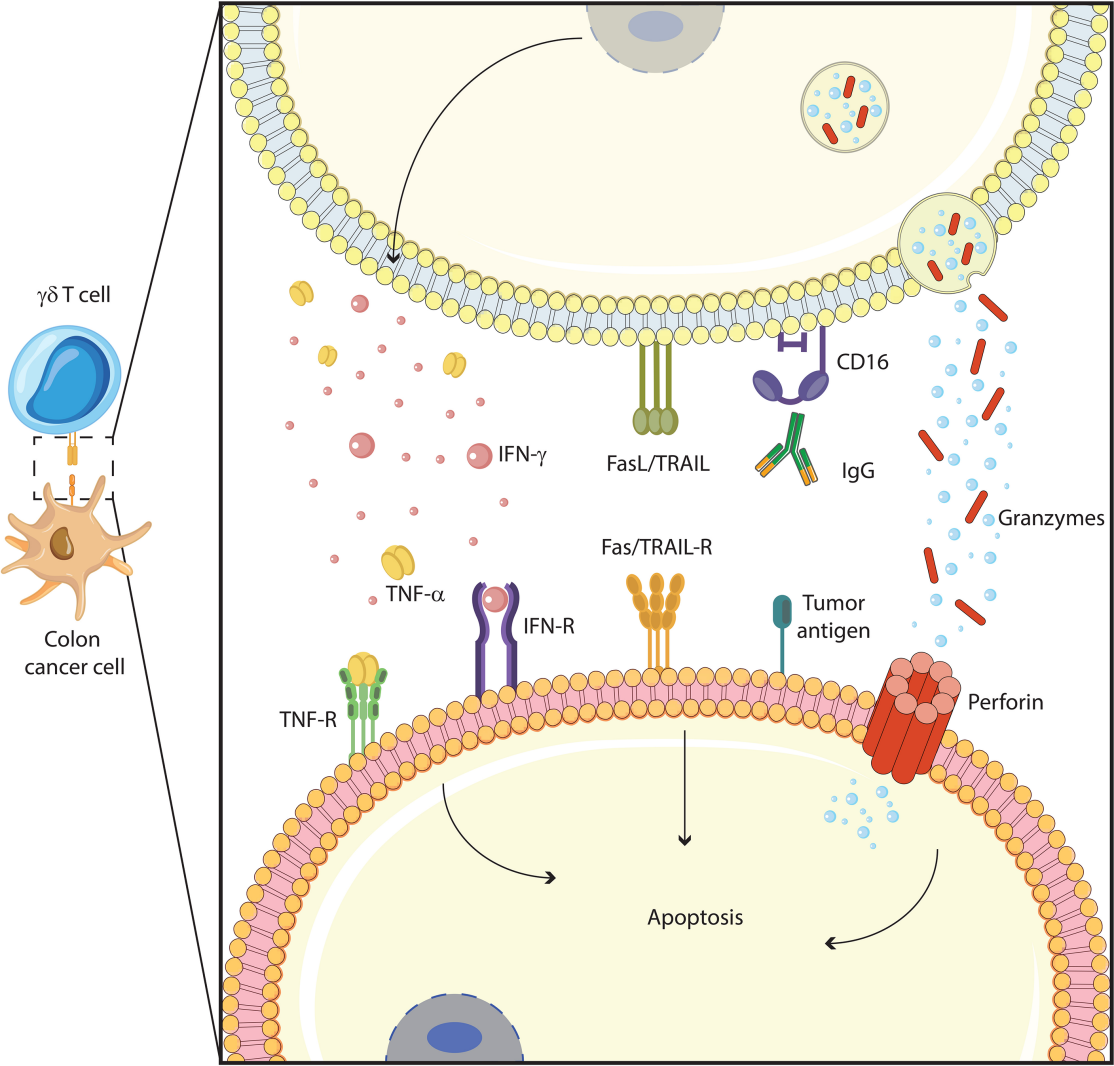
γδ T cells function against colon cancer cells. γδ T cells exert their cytotoxic functions through several mechanisms: induction of apoptotic cell death with Fas/TRAIL pathway, antibody-dependent cellular cytotoxicity (ADCC) with IgG/CD16 interaction, cytolytic granule (granzyme, perforin) polarization and degranulation, and proinflammatory cytokines (TNF-α, IFN-γ) production.

γδ T cells, particularly Vδ1 and Vδ2 subsets, have a significant antitumor activity that is exerted by direct and indirect mechanisms and through the involvement of several pathways. Cancer cells are recognized not only *via* TCR-dependent antigen recognition but also through NK receptors and or antibody-dependent cell-mediated cytotoxicity (ADCC) (**Figures 1**, **2**).

Vγ9Vδ2 T cells generally recognize tumor target cells either through the TCR or through stimulatory NK receptors, such as NKG2D and DNAX accessory molecule 1 (DNAM-1), that recognize their specific ligands overexpressed on tumor cells. In addition to NKG2D, Vδ1^+^ T cells used natural cytotoxicity receptors (NKp30, NKp44, NKp46) for tumor cell recognition, although their expression is linked to specific conditions, such as the presence of IL-15 or IL-2 (12, 53). A recent study shows the strong antitumor activity of NKp46^+^ γδ IELs against human colon adenocarcinoma cell lines (12).

Similar to NK cells, human γδ T cells, once activated, upregulate on their surface type-III Fcγ receptor (CD16) and promote ADCC on opsonized tumor cells. Relevant to cancer immunotherapy, CD16-expressing γδ T cells have been shown to improve the efficacy of therapeutic antibodies such as rituximab and trastuzumab for the treatment of lymphoma, chronic lymphocytic leukemia, and human epidermal growth factor receptor 2 (HER2)-positive breast cancer cells (54, 55). Recent studies have shown that bispecific antibodies binding TCR-HER2 and CD19-specific triple bodies increase γδ T cells cytotoxicity, improving ADCC (56) (57),. Finally, Vγ9Vδ2 T lymphocytes with high CD16 expression exert ADCC in the 3D cultures of colon cancer cells, inducing a decrease in size and cell number in spheroids (58). Whatever the mechanism of target cell recognition, killing by γδ T cells involves the perforin/granzyme pathways (59, 60) and Fas/FasL, TRAIL/TRAIL-R, and TNF/TNF-RII interactions (61–63), depending on the nature of the target cells.

Instead, indirect antitumor mechanisms occur *via* interaction between γδ T cells and other immune cells, such as B cells, DC, αβ T cells, and NK cells. Acting similarly to Tfh cells, γδ T cells can secrete cytokines that promote B cells to secrete specific antibodies (29). Furthermore, they take part in the maturation of DC, thus inducing helper and cytotoxic functions of CD4^+^ and CD8^+^ T cells (64, 65). Likewise, they improve NK cell-mediated cytotoxicity through CD137/CD137L engagement and upregulating NKG2D (66).

## How to use γδ T cells for immunotherapeutic protocols

In the last few years, immunotherapy has had great prominence in cancer treatment. It is based on enhancing the immune system to kill tumor cells and block or limit their growth. Most immunotherapeutic approaches make use of αβ T cells, but efficacy is limited due to the characteristics of this subset, namely, their activation and functional activities rely on MHC restriction, costimulatory signal, and specific tumor-associated antigen (TAA) recognition (67).

γδ T cells provide themselves with a valid alternative to overcome these limits thanks to their unique features (68). Indeed, they recognize conserved antigens widely expressed on stressed and tumor cells without the need for MHC restriction and costimulatory molecules. Both of these aspects allow γδ T cells to avoid immune escape due to the downregulation of MHC molecules and loss of antigen expression by tumor cells. In addition, they perform effector activity relevant to antitumor immune responses, such as cytotoxicity and cytokine (IFN-γ and TNF-α) production. Finally, their inbred tissue tropism, particularly for Vδ1^+^ T cells, makes them more suitable than αβ T cells for performing rapid antitumor responses. These features, together with their easy activation and expansion, make γδ T cells attractive candidates for tumor immunotherapy (69).

**Figure 3:**
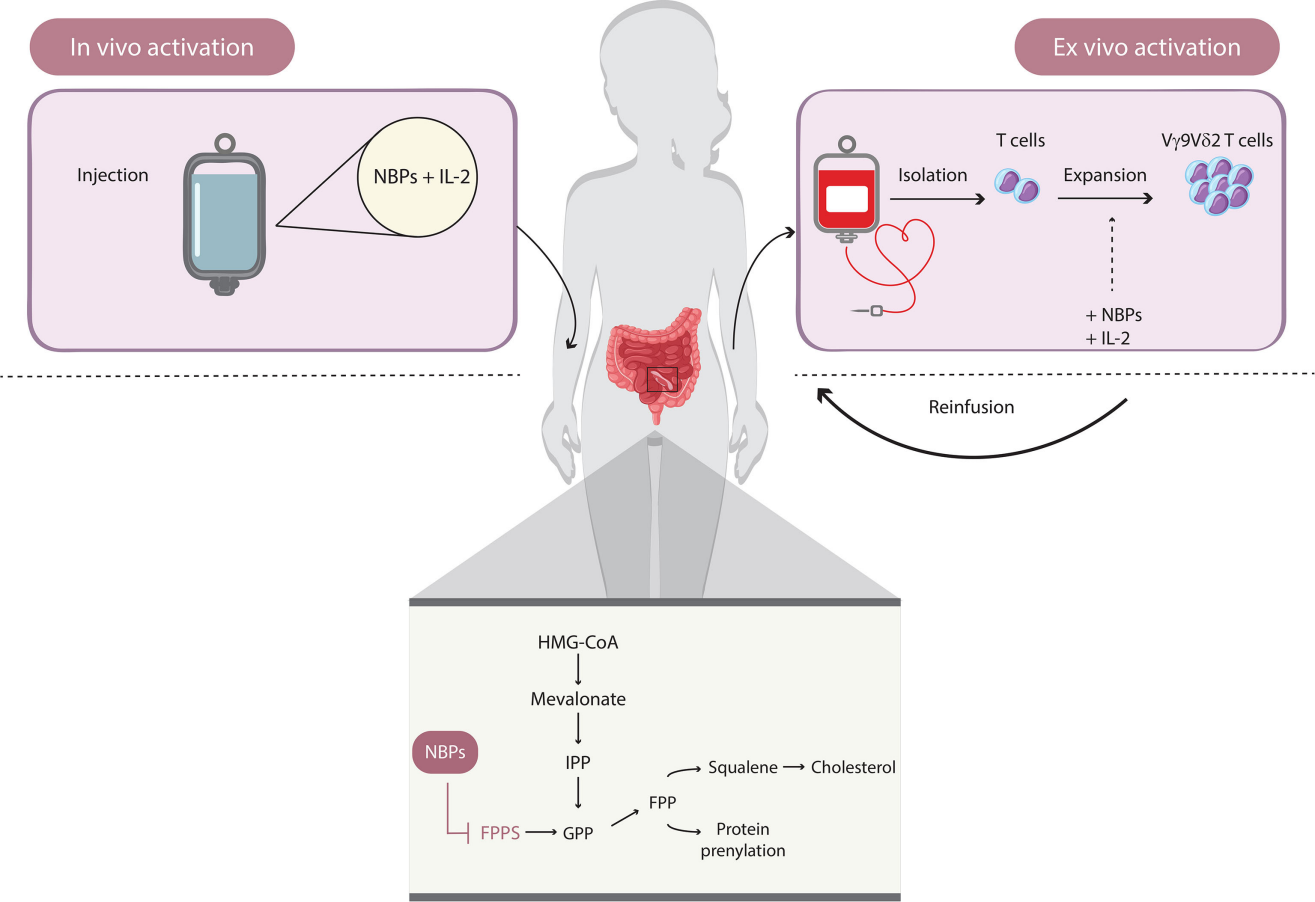
How to use γδ T cells in immunotherapy. Immunotherapeutic approaches based on Vγ9Vδ2 T cells involve two types of mechanisms: *in vivo* activation and adoptive transfer of *ex vivo*-expanded Vγ9Vδ2 T cells. The *in vivo* activation requires the administration of amino bisphosphonates (e.g., zoledronate), which induce the accumulation of endogenous phosphoantigens (P-Ags; such as IPP) by inhibiting the farnesyl pyrophosphate synthase of the mevalonate pathway and upregulating isoprenoid biosynthesis within cells. The *ex vivo* approach is based on the isolation of γδ T cells from peripheral blood mononuclear cells and the activation of Vγ9Vδ2 T cell, induced directly by natural (e.g., IPP and HMBPP) or synthetic (e.g., BrHPP) P-Ags plus IL-2 and the adoptive transfer.

Most γδ T-cell-based immunotherapy clinical trials have used the Vγ9Vδ2 subset (**Figure 3**). According to the modality of their expansion and activation, two types of Vγ9Vδ2 T-cell immunotherapy have been considered: *in vivo* activation and adoptive transfer of *ex vivo* expanded Vγ9Vδ2 T cells (17). The *ex vivo* approach requires the isolation of γδ T cells from peripheral blood mononuclear cells (PBMCs), their expansion *in vitro*, and then their adoptive transfer into recipient patients. Specific stimulants for Vγ9Vδ2 T cells include natural (IPP and HMBPP) or synthetic (bromohydrin pyrophosphate (BrHPP)) P-Ags. Furthermore, different drugs can be used to enhance the accumulation of endogenous P-Ags, among which are nitrogenous bisphosphonates (N-BPs), a class of drugs generally used in the treatment of osteoporosis and bone disorders. They act by inhibiting the farnesyl pyrophosphate synthase (FPPS) enzyme of the mevalonate pathway, thus upregulating isoprenoid biosynthesis within cells (70). Zoledronic acid (zoledronate, Zometa (ZA)) is the most common *in vivo* and *in vitro* using N-BP and it also shows antitumor effects through induction of apoptosis and autophagy in cancer cell lines (71).

Another alternative approach is the *in vivo* activation of Vγ9Vδ2 T cells by the administration of P-Ags or N-BPs in combination with IL-2, which is necessary for Vγ9Vδ2 expansion (72). This strategy increases with success the number of circulating Vγ9Vδ2, but it does not ensure their recruitment to the target tissue, where they can exert antitumor functions.

However, it has been shown both in humans and in nonhuman primate models that, upon repeated injections of P-Ags, Vγ9Vδ2 do not respond to P-Ags, probably because of activation-induced energy or exhaustion of their functions, promoting terminal differentiation (73).

In light of these limits, Vδ1^+^ T cells apply for a possible alternative to or support to Vγ9Vδ2-based immunotherapy. Indeed, two studies conducted on primary malignant melanoma and colorectal cancer pinpointed Vδ1^+^ T cells as the major γδ T-cell subset among TILs (37) (39),. Indeed, as well as showing a higher cytotoxic capacity compared to Vδ2, Vδ1^+^ T cells have the advantage of tissue residency, so the adoptive transfer of Vδ1 may be efficient in reaching the tumor site. Once activated by a broad variety of ligands, Vδ1^+^ T cells are also able to circulate and persist for a long time in the body, promoting long-term therapeutic effects. This is probably due to the fact that they are resistant to activation-induced cell death (74). Nevertheless, their clinical application is difficult due to the lack of a well-established expansion protocol. Recently, one of the protocols developed that has given promising results is established by Almeida et al., in which they expanded Vδ1^+^ from the peripheral blood of healthy donors and patients with chronic lymphocytic leukemia (CLL). These cells differentiate into a highly cytotoxic subset and inhibit tumor growth and metastasis in xenograft models of CLL *in vivo* (53). Based on this expansion procedure, GammaDelta Therapeutics has developed an allogeneic, nonengineered Vδ1 T-cell therapy (GDX012) for the treatment of patients with acute myeloid leukemia with minimal residual disease (NCT05001451) (75).

The newest γδ T-cell-based therapeutic strategies include the use of bispecific γδ T-cell engagers and γδ CAR-T cells (76). Vγ9-TCR-specific engagers binding to either CD123 (77) or HER2 (56) mediate cytotoxicity against tumor cells in acute myeloid leukemia and pancreatic adenocarcinomas, respectively. Moreover, they have the potential to enhance the efficacy of adoptively transferred γδ T cells (78). Lava Therapeutics is currently conducting two open-label phase I/IIa studies to assess the efficacy and safety of LAVA-051 (79), a humanized bispecific single-domain antibody that directly engages CD1d and the Vδ2 chain of Vγ9Vδ2 T cells in patients with relapsed/refractory chronic lymphocytic leukemia, multiple myeloma, and acute myeloid leukemia (NCT04887259) and LAVA-1207, which activates Vγ9Vδ2 T cells upon crosslinking to prostate-specific membrane antigen (PSMA) in patients with metastatic castration-resistant prostate cancer (NCT05369000).

In addition to Vγ9Vδ2 T cells, recently γδ CAR-T-cell-based therapy is also based on genetically modified Vδ1 T cells (80, 81). Adicet Bio is currently conducting a phase I clinical trial on anti-CD20 allogeneic γδ CAR-T cells for patients with B-cell malignancies (NCT04735471) (81). Compared to αβ CAR-T, the autologous or allogenic Vδ1/Vδ2 T-cell-expressing CAR ensures a reduced risk of a cytokine-related syndrome and a lower risk of off-target toxicity, as well as a long lifespan and preserved ability to act as antigen-presenting cells (82).

## γδ T-cell-based CRC immunotherapy

A growing body of evidence has allowed us to understand the role of γδ T cells in the tumor milieu and pave the way for their potential use in new anti-CRC immunotherapy approaches. CRC immunotherapy based on γδ T cells has only produced a small number of clinical results so far, although it has proven to be safe in terms of tolerability and side effects.

In a clinical trial with six CRC patients who had undergone lung metastasectomy, Izumi and colleagues used an injection of autologous Vγ9Vδ2 T cells, expanded with 5 μM of ZA and 1,000 IU/ml of IL-2, for 8 weeks. During treatment, the number of Vγ9Vδ2 T cells increased and remained stable long after the last infusions in terms of percentage and absolute number. Moreover, Vγ9Vδ2 T cells expressed more IFN-γ and CD107a *ex vivo* than other Vγ9^−^ CD3^+^ T cells (83). In a phase I trial, Bennouna et al. tested *in vivo* the pharmacodynamic profile of γδ-agonist, BrHPP (IPH1101), in 28 patients with solid tumors, among which there were three CRC patients. They first evaluated the maximum-tolerated dose of BrHPP administered alone during the first cycle. Because the expansion and activation of Vγ9Vδ2 T lymphocytes required IL-2, they combined BrHPP with a low dose of IL-2. This approach proved to be safe, generally well-tolerated, and induced a strong γδ T lymphocyte expansion in these patients (84). Noguchi et al. performed a clinical trial enrolling 25 patients with advanced solid tumors, one of them with colon cancer. Once stimulated and expanded *ex vivo* with ZA and IL-2, γδ T cells were reinfused into patients. This approach did not cause severe toxicity, but rather restored the number of effector γδ T cells (85). Another phase I clinical study, including three patients with CRC, tested the adoptive transfer of *ex vivo* expanded autologous Vγ9Vδ2 T cells to evaluate whether the approach was doable and safe. No dose-dependent toxicity was found, but for some patients, the therapy was ineffective (86).

It is known that colon cancer is generated by a small population of cancer stem cells, which are relevant players in tumor persistence and drug resistance. Colon cancer stem cells are resistant to Vγ9Vδ2 T-cell cytotoxicity, but when sensitized with ZA (or even chemotherapeutic agents), they are efficiently killed by Vγ9Vδ2 T cells (60, 87). However, due to the biodistribution of ZA, including its bone tropism, its use *in vivo* can often be ineffective in properly reaching and stimulating γδ T lymphocytes in tissues. In this regard, recent work has shown that it is possible to encapsulate ZA in spherical polymeric nanoparticles (SPNs), which, thanks to the enhanced permeability and retention effect, can easily reach the tumor site. ZA-SPNs are engulfed by CRC cells, and Vδ2^+^ T cells interact with ZA-SPN-treated CRC cells, promoting their killing. In addition, Vδ2^+^ T cells also kill ZA-SPN-treated CRC tumor spheroids and autologous tumor organoids (88).

Ang et al. expanded Vγ9Vδ2 T cells with ZA/IL2 and constructed NKG2D ligand-specific chimeric antigen receptors (NKG2DL) using mRNA electroporation. Upon interaction with NKG2DL^+^ cancer cells, the CAR-γδ T cells enhanced their cytotoxic activity against a variety of cultured solid tumor cell lines, including those resistant to ZA treatment. They also evaluated the *in vivo* tumor-killing effect of the NKG2Dz RNA CAR-modified Vγ9Vδ2 T cells in a colorectal cancer mouse model using repeated intraperitoneal injections of aggressive human colorectal cancer cell lines. NKG2Dz RNA CAR-modified Vγ9Vδ2 T cells significantly slowed tumor progression, resulting in a median survival time of 57 days (89). Gadeta’s second cell therapy product, GDT201, which combines HLA-independent tumor recognition capabilities of γδ TCRs with proliferative capacity and robust tumor killing of αβ T cells, demonstrated long-lasting cytotoxicity effect on CRC cell lines, patient-derived tumor cells, and in a xenograft tumor model, suggesting the potential to address well-known clinical hurdles such as lack of tumor restricted antigens and safety (90).

As the functions of γδ T lymphocytes are often switched off by the microenvironment, a further useful approach would be to combine their expansion with the use of inhibitors of their negative regulators, thus restoring their ability to mediate protective immunity against cancer cells. Lu et al. have shown a high expression of B7-H3, an important member of the B7 superfamily, on CRC-infiltrating Vδ2^+^ T cells. B7-H3 appears to be an important negative regulator of their Vδ2^+^ T-cell antitumor functions, specifically *via* the downregulation of IFN-γ and perforin/granzyme B expression. Indeed, by inhibiting B7-H3 with a specific blocking antibody (MIH35) or B7-H3 siRNA, they witness the reactivation of effector functions, a decrease of apoptosis rates, and an upregulation of the activation markers CD25 and CD69. These treatments promoted the cytotoxic activity of Vδ2^+^ T cells on colon cancer cell lines. Hereupon, this suggests that it would be useful to combine *in vivo* activation of Vδ2^+^ T cells with the inhibition or blockade of B7-H3 as another immunotherapeutic approach for colon cancer (91).

Tim-3 is another inhibitory receptor that can influence the activity of γδ T lymphocytes. Its signaling significantly hindered the killing activity of Vγ9Vδ2 T cells against colon cancer cells and reduced the expression of perforin and granzyme B cells in an ERK1/2-dependent manner (92); because γδ T cells express high levels of Tim-3 in peripheral blood and colon cancer tissue, Tim-3 blockade by neutralizing antibody restores their cytotoxicity towards colon cancer cells. Consistent with this strategy, the inhibition of glycogen synthase kinase-3β with TWS119 inhibitor, boost proliferation, differentiation, and cytolytic activity of γδ T cells against colon cancer cells, activating mammalian target rapamycin (mTOR) pathway and upregulating the antiapoptotic protein Bcl-2 as well as the expression of perforin and granzyme B *in vitro* and *in vivo* (59).

Exploiting γδ T cells as a therapeutic tool can also be beneficial to overcome the failure of immunotherapies caused by the mutational state of the tumor. In this regard, de Bruin et al. generated and applied *in vitro* and *in vivo* a bispecific nanobody-based construct that, on the one hand, inhibited the signaling of EGFR and, on the other, induced target-dependent activation of Vγ9Vδ2 T cells. When co-cultured with two different EGFR^+^ human colon cancer cell lines, each with KRAS and BRAF mutations, and in the presence of the bispecific construct, Vγ9Vδ2 T cells produced IFN-γ and TNF-α and induced lysis of both cancer cell lines, irrespective of their mutational state. The same effects were found *in vivo*, combining the administration of Vγ9Vδ2 T cells with the bispecific construct (93).

Although most immunotherapy protocols are based on Vγ9Vδ2 T cells, promising results have been achieved using Vδ1^+^ T cells. After their isolation from PBMC and expansion with PHA and IL-7, Vδ1^+^ T cells were performed *in vitro* with strong cytotoxic and cytolytic effects against adherent and sphere-forming human colon cancer cells in a contact-dependent manner. *Ex vivo* expanded Vδ1^+^ T cells were able to block tumor growth in a CRC xenograft model (94). Furthermore, in another murine model of metastatic colon cancer obtained from orthotropic implantation of human HT29 cells, the administration of CMV-induced Vδ1^+^ T cells suppressed the progression of the primary tumor and the onset of metastasis (95).

## Conclusions

CRC is a very complex and multifactorial tumor, characterized by a high mutation burden. Due to these features, standard treatment regimens have been proven to be ineffective in many patients. Here comes immunotherapy into play, especially targeting the checkpoint blockade pathway to enhance antitumor responses, although so far in CRC it does not seem to be always applicable compared to other solid tumors that respond well. Indeed, in MSI-H/dMMR CRC, this therapeutic approach is useful, whereas the majority of MSS/pMMR CRC patients do not benefit from immunotherapy (2). Hence, new immunotherapeutic approaches are needed, taking advantage of the interactions between tumors and the immune system. In recent years, the role of γδ T lymphocytes has emerged as an excellent therapeutic platform in hematological and solid tumors, including CRC. This is made possible by their hallmarks, which make them easily available, manipulable, and safe. Furthermore, to improve their immunogenicity and infiltration at the tumor sites in CRC, a combination of different strategies has been recently suggested, although their clinical relevance has yet to be evaluated. Little is known about the nature of the antigens and contacts that activate Vδ1 T cells, and a deeper understanding of their biology is required to maximize their therapeutic potential. Despite the beneficial aspects of γδ-based immunotherapy, clinical trials for CRC are still in their early stages. Therefore, future colon cancer immunotherapy could benefit from new engineering technologies, such as CAR-γδ T cells (96) and γδ T-cell-based bispecific constructs (97), to improve their antitumor efficacy.

## Author contributions

AC, MDS, and SM designed the entire review. AC and MDS wrote the paper. ELP, FD, and SM reviewed and edited the manuscript. All authors contributed to the article and approved the submitted version.

## References

[1] Bray F Ferlay J Soerjomataram I Siegel RL Torre LA Jemal A. Global cancer statistics 2018: GLOBOCAN estimates of incidence and mortality worldwide for 36 cancers in 185 countries. CA: Cancer J Clin (2018) 68(6):394–424. doi: 10.3322/caac.21492 30207593

[2] Lichtenstern CR Ngu RK Shalapour S Karin M. Immunotherapy, inflammation and colorectal cancer. Cells. (2020) 9(3):618. doi: 10.3390/cells9030618 32143413 PMC7140520

[3] Yamagishi H Kuroda H Imai Y Hiraishi H. Molecular pathogenesis of sporadic colorectal cancers. Chin J Cancer. (2016) 35:4–. doi: 10.1186/s40880-015-0066-y PMC4704376 26738600

[4] Siegel RL Miller KD Fedewa SA Ahnen DJ Meester RGS Barzi A. Colorectal cancer statistics, 2017. CA: Cancer J Clin (2017) 67(3):177–93. doi: 10.3322/caac.21395 28248415

[5] Ganesh K Stadler ZK Cercek A Mendelsohn RB Shia J Segal NH. Immunotherapy in colorectal cancer: Rationale, challenges and potential. Nat Rev Gastroenterol hepatology. (2019) 16(6):361–75. doi: 10.1038/s41575-019-0126-x PMC7295073 30886395

[6] Galon J Costes A Sanchez-Cabo F Kirilovsky A Mlecnik B Lagorce-Pagès C. Type, density, and location of immune cells within human colorectal tumors predict clinical outcome. Sci (New York NY). (2006) 313(5795):1960–4. doi: 10.1126/science.1129139 17008531

[7] Xie Y-H Chen Y-X Fang J-Y. Comprehensive review of targeted therapy for colorectal cancer. Signal Transduction Targeted Ther (2020) 5(1):22. doi: 10.1038/s41392-020-0116-z PMC7082344 32296018

[8] Wu T Dai Y. Tumor microenvironment and therapeutic response. Cancer letters. (2017) 387:61–8. doi: 10.1016/j.canlet.2016.01.043 26845449

[9] Pagès F Mlecnik B Marliot F Bindea G Ou FS Bifulco C. International validation of the consensus immunoscore for the classification of colon cancer: A prognostic and accuracy study. Lancet (London England). (2018) 391(10135):2128–39. doi: 10.1016/S0140-6736(18)30789-X 29754777

[10] Lo Presti E Pizzolato G Corsale AM Caccamo N Sireci G Dieli F. γδ T cells and tumor microenvironment: From immunosurveillance to tumor evasion. Front Immunol (2018) 9:1395. doi: 10.3389/fimmu.2018.01395 29963061 PMC6013569

[11] Nielsen MM Witherden DA Havran WL. γδ T cells in homeostasis and host defence of epithelial barrier tissues. Nat Rev Immunol (2017) 17(12):733–45. doi: 10.1038/nri.2017.101 PMC5771804 28920588

[12] Mikulak J Oriolo F Bruni E Roberto A Colombo FS Villa A. NKp46-expressing human gut-resident intraepithelial Vδ1 T cell subpopulation exhibits high antitumor activity against colorectal cancer. JCI Insight (2019) 4(24):e125884. doi: 10.1172/jci.insight.125884 31689241 PMC6975269

[13] Buccheri S Guggino G Caccamo N Li Donni P Dieli F. Efficacy and safety of γδT cell-based tumor immunotherapy: A meta-analysis. J Biol regulators homeostatic agents. (2014) 28(1):81–90. 24750794

[14] Vantourout P Hayday A. Six-of-the-best: Unique contributions of γδ T cells to immunology. Nat Rev Immunol (2013) 13(2):88–100. doi: 10.1038/nri3384 23348415 PMC3951794

[15] Garcillán B Marin AVM Jiménez-Reinoso A Briones AC Muñoz-Ruiz M García-León MJ. Gd T lymphocytes in the diagnosis of human T cell receptor immunodeficiencies. Front Immunol (2015) 6:20. doi: 10.3389/fimmu.2015.00020 25688246 PMC4310324

[16] Fichtner AS Ravens S Prinz I. Human γδ TCR repertoires in health and disease. Cells. (2020) 9(4):800. 32225004 10.3390/cells9040800 PMC7226320

[17] Yazdanifar M Barbarito G Bertaina A Airoldi I. γδ T cells: The ideal tool for cancer immunotherapy. Cells. (2020) 9(5):1305. doi: 10.3390/cells9051305 32456316 PMC7290982

[18] Xu B Pizarro JC Holmes MA McBeth C Groh V Spies T. Crystal structure of a γδ T-cell receptor specific for the human MHC class I homolog MICA. PNAS (2011) 201015433:2414–9. doi: 10.1073/pnas.1015433108 PMC3038733 21262824

[19] Catellani S Poggi A Bruzzone A Dadati P Ravetti J Gobbi M. Expansion of Vδ1 T lymphocytes producing IL-4 in low-grade non-Hodgkin lymphomas expressing UL-16-binding proteins. Blood. (2007) 109:2078–85. doi: 10.1182/blood-2006-06-028985 16973957

[20] Uldrich AP Le Nours J Pellicci DG Gherardin NA McPherson KG Lim RT. CD1d-lipid antigen recognition by the γδ TCR. Nat Immunol (2013) 14(11):1137–45. doi: 10.1038/ni.2713 24076636

[21] Luoma AM Castro CD Mayassi T Bembinster LA Bai L Picard D. Crystal structure of Vδ1 T cell receptor in complex with CD1d-sulfatide shows MHC-like recognition of a self-lipid by human γδ T cells. Immunity. (2013) 39(6):1032–42. doi: 10.1016/j.immuni.2013.11.001 PMC3875342 24239091

[22] Khairallah C Déchanet-Merville J Capone M. γδ T cell-mediated immunity to cytomegalovirus infection. Front Immunol (2017) 8:105–. doi: 10.3389/fimmu.2017.00105 PMC5298998 28232834

[23] Sant S Jenkins MR Dash P Watson KA Wang Z Pizzolla A. Human γδ T-cell receptor repertoire is shaped by influenza viruses, age and tissue compartmentalisation. Clin Transl Immunol (2019) 8(9):e1079–e. doi: 10.1002/cti2.1079 PMC6756999 31559018

[24] Hayday AC. [gamma][delta] cells: A right time and a right place for a conserved third way of protection. Annu Rev Immunol (2000) 18:975–1026. doi: 10.1146/annurev.immunol.18.1.975 10837080

[25] Gogoi D Chiplunkar SV. Targeting gamma delta T cells for cancer immunotherapy: Bench to bedside. Indian J Med Res (2013) 138(5):755–61. PMC3928706 24434328

[26] Gu S Nawrocka W Adams EJ. Sensing of pyrophosphate metabolites by Vγ9Vδ2 T cells. Front Immunol (2014) 5:688. doi: 10.3389/fimmu.2014.00688 25657647 PMC4303140

[27] De Rosa SC Andrus JP Perfetto SP Mantovani JJ Herzenberg LA Herzenberg LA. Ontogeny of γδ T cells in humans. J Immunol (2004) 172(3):1637–45. doi: 10.4049/jimmunol.172.3.1637 14734745

[28] Rigau M Ostrouska S Fulford TS Johnson DN Woods K Ruan Z. Butyrophilin 2A1 is essential for phosphoantigen reactivity by γδ T cells. Sci (New York NY) (2020) 367(6478):eaay5516. doi: 10.1126/science.aay5516 31919129

[29] Caccamo N Battistini L Bonneville M Poccia F Fournié JJ Meraviglia S. CXCR5 identifies a subset of Vgamma9Vdelta2 T cells which secrete IL-4 and IL-10 and help b cells for antibody production. J Immunol (2006) 177(8):5290–5. doi: 10.4049/jimmunol.177.8.5290 17015714

[30] Caccamo N Sireci G Meraviglia S Dieli F Ivanyi J Salerno A. γδ T cells condition dendritic cells in vivo for priming pulmonary CD8 T cell responses against mycobacterium tuberculosis. Eur J Immunol (2006) 36(10):2681–90. doi: 10.1002/eji.200636220 16981183

[31] Davey MS Willcox CR Hunter S Kasatskaya SA Remmerswaal EBM Salim M. The human Vδ2(+) T-cell compartment comprises distinct innate-like Vγ9(+) and adaptive Vγ9(-) subsets. Nat Commun (2018) 9(1):1760. doi: 10.1038/s41467-018-04076-0 29720665 PMC5932074

[32] Mangan BA Dunne MR O’Reilly VP Dunne PJ Exley MA O’Shea D. Cutting edge: CD1d restriction and Th1/Th2/Th17 cytokine secretion by human Vδ3 T cells. J Immunol (2013) 191(1):30–4. doi: 10.4049/jimmunol.1300121 PMC3721026 23740951

[33] Petrasca A Melo AM Breen EP Doherty DG. Human Vδ3(+) γδ T cells induce maturation and IgM secretion by b cells. Immunol letters. (2018) 196:126–34. doi: 10.1016/j.imlet.2018.02.002 29438730

[34] Willcox CR Pitard V Netzer S Couzi L Salim M Silberzahn T. Cytomegalovirus and tumor stress surveillance by binding of a human γδ T cell antigen receptor to endothelial protein c receptor. Nat Immunol (2012) 13(9):872–9. doi: 10.1038/ni.2394 22885985

[35] Silva-Santos B Serre K Norell H. γδ T cells in cancer. Nat Rev Immunol (2015) 15(11):683–91. doi: 10.1038/nri3904 26449179

[36] Hidalgo JV Bronsert P Orlowska-Volk M Díaz LB Stickeler E Werner M. Histological analysis of γδ T lymphocytes infiltrating human triple-negative breast carcinomas. Front Immunol (2014) 5:[632 p.]. [Internet]. doi: 10.3389/fimmu.2014.00632 PMC4261817 25540645

[37] Cordova A Toia F La Mendola C Orlando V Meraviglia S Rinaldi G. Characterization of human γδ T lymphocytes infiltrating primary malignant melanomas. PloS One (2012) 7(11):e49878. doi: 10.1371/journal.pone.0049878 23189169 PMC3506540

[38] Kakimi K Matsushita H Murakawa T Nakajima J. γδ T cell therapy for the treatment of non-small cell lung cancer. Transl Lung Cancer Res (2014) 3(1):23–33. doi: 10.3978/j.issn.2218-6751.2013.11.01 25806278 PMC4367606

[39] Meraviglia S Lo Presti E Tosolini M La Mendola C Orlando V Todaro M. Distinctive features of tumor-infiltrating γδ T lymphocytes in human colorectal cancer. Oncoimmunology. (2017) 6(10):e1347742. doi: 10.1080/2162402X.2017.1347742 29123962 PMC5665062

[40] Corvaisier M Moreau-Aubry A Diez E Bennouna J Mosnier JF Scotet E. V Gamma 9V delta 2 T cell response to colon carcinoma cells. J Immunol (2005) 175(8):5481–8. doi: 10.4049/jimmunol.175.8.5481 16210656

[41] de Vries NL van Unen V Ijsselsteijn ME Abdelaal T van der Breggen R Farina Sarasqueta A. High-dimensional cytometric analysis of colorectal cancer reveals novel mediators of antitumour immunity. Gut. (2020) 69(4):691. doi: 10.1136/gutjnl-2019-318672 31270164 PMC7063399

[42] Lo Presti E Corsale AM Di Simone M Dieli F Meraviglia S. Characterisation of γδ T cells infiltrating colorectal cancer. Gut. (2021) 70(5):1001–3. doi: 10.1136/gutjnl-2020-322101 32737063

[43] Zhao Y Niu C Cui J. Gamma-delta (γδ) T cells: Friend or foe in cancer development? J Trans Med (2018) 16(1):3. doi: 10.1186/s12967-017-1378-2 PMC5761189 29316940

[44] Gentles AJ Newman AM Liu CL Bratman SV Feng W Kim D. The prognostic landscape of genes and infiltrating immune cells across human cancers. Nat Med (2015) 21(8):938–45. doi: 10.1038/nm.3909 PMC4852857 26193342

[45] Tosolini M Pont F Poupot M Vergez F Nicolau-Travers ML Vermijlen D. Assessment of tumor-infiltrating TCRVγ9Vδ2 γδ lymphocyte abundance by deconvolution of human cancers microarrays. Oncoimmunology. (2017) 6(3):e1284723. doi: 10.1080/2162402X.2017.1284723 28405516 PMC5384348

[46] Wu P Wu D Ni C Ye J Chen W Hu G. γδT17 cells promote the accumulation and expansion of myeloid-derived suppressor cells in human colorectal cancer. Immunity. (2014) 40(5):785–800. doi: 10.1016/j.immuni.2014.03.013 24816404 PMC4716654

[47] Xiang Z Tu W. Dual face of Vγ9Vδ2-T cells in tumor immunology: Anti- versus pro-tumoral activities. Front Immunol (2017) 8:1041. doi: 10.3389/fimmu.2017.01041 28894450 PMC5581348

[48] Patil RS Shah SU Shrikhande SV Goel M Dikshit RP Chiplunkar SV. IL17 producing γδT cells induce angiogenesis and are associated with poor survival in gallbladder cancer patients. Int J Cancer. (2016) 139(4):869–81. doi: 10.1002/ijc.30134 27062572

[49] Chen X Shang W Xu R Wu M Zhang X Huang P. Distribution and functions of γδ T cells infiltrated in the ovarian cancer microenvironment. J Trans Med (2019) 17(1):144. doi: 10.1186/s12967-019-1897-0 PMC6505080 31064389

[50] Rong L Li K Li R Liu H-M Sun R Liu X-Y. Analysis of tumor-infiltrating gamma delta T cells in rectal cancer. World J Gastroenterol (2016) 22(13):3573–80. doi: 10.3748/wjg.v22.i13.3573 PMC4814643 27053849

[51] Hu G Wu P Cheng P Zhang Z Wang Z Yu X. Tumor-infiltrating CD39(+)γδTregs are novel immunosuppressive T cells in human colorectal cancer. Oncoimmunology. (2017) 6(2):e1277305. doi: 10.1080/2162402X.2016.1277305 28344891 PMC5353931

[52] Chabab G Barjon C Abdellaoui N Salvador-Prince L Dejou C Michaud HA. Identification of a regulatory Vδ1 gamma delta T cell subpopulation expressing CD73 in human breast cancer. J leukocyte Biol (2020) 107(6):1057–67. doi: 10.1002/JLB.3MA0420-278RR 32362028

[53] Almeida AR Correia DV Fernandes-Platzgummer A da Silva CL da Silva MG Anjos DR. Delta one T cells for immunotherapy of chronic lymphocytic leukemia: Clinical-grade Expansion/Differentiation and preclinical proof of concept. Clin Cancer Res an Off J Am Assoc Cancer Res (2016) 22(23):5795–804. doi: 10.1158/1078-0432.CCR-16-0597 27307596

[54] Tokuyama H Hagi T Mattarollo SR Morley J Wang Q Fai-So H. Vγ9Vδ2 T cell cytotoxicity against tumor cells is enhanced by monoclonal antibody drugs–rituximab and trastuzumab. Int J Cancer. (2008) 122(11):2526–34. doi: 10.1002/ijc.23365 18307255

[55] Capietto A-H Martinet L Fournié J-J. Stimulated γδ T cells increase the *In vivo* efficacy of trastuzumab in HER-2+ breast cancer. J Immunol (2011) 187(2):1031–8. doi: 10.4049/jimmunol.1100681 21670311

[56] Oberg HH Peipp M Kellner C Sebens S Krause S Petrick D. Novel bispecific antibodies increase γδ T-cell cytotoxicity against pancreatic cancer cells. Cancer Res (2014) 74(5):1349–60. doi: 10.1158/0008-5472.CAN-13-0675 24448235

[57] Schiller CB Braciak TA Fenn NC Seidel UJE Roskopf CC Wildenhain S. CD19-specific triplebody SPM-1 engages NK and γδ T cells for rapid and efficient lysis of malignant b-lymphoid cells. Oncotarget. (2016) 7(50):83392–408. doi: 10.18632/oncotarget.13110 PMC5347777 27825135

[58] Varesano S Zocchi MR Poggi A. Zoledronate triggers Vδ2 T cells to destroy and kill spheroids of colon carcinoma: Quantitative image analysis of three-dimensional cultures. Front Immunol (2018) 9:998–. doi: 10.3389/fimmu.2018.00998 PMC5951939 29867975

[59] Chen YQ Zheng L Aldarouish M Zhou ZH Pan N Liu JQ. Wnt pathway activator TWS119 enhances the proliferation and cytolytic activity of human γδT cells against colon cancer. Exp Cell Res (2018) 362(1):63–71. doi: 10.1016/j.yexcr.2017.11.003 29104081

[60] Todaro M D'Asaro M Caccamo N Iovino F Francipane MG Meraviglia S. Efficient killing of human colon cancer stem cells by gammadelta T lymphocytes. J Immunol (2009) 182(11):7287–96. doi: 10.4049/jimmunol.0804288 19454726

[61] Dalton JE Howell G Pearson J Scott P Carding SR. Fas-fas ligand interactions are essential for the binding to and killing of activated macrophages by gamma delta T cells. J Immunol (2004) 173(6):3660–7. doi: 10.4049/jimmunol.173.6.3660 15356111

[62] Todaro M D'Asaro M Caccamo N Iovino F Francipane MG Meraviglia S. Efficient killing of human colon cancer stem cells by γδ T lymphocytes. J Immunol (2009) 182(11):7287–96. doi: 10.4049/jimmunol.0804288 19454726

[63] Ramstead AG Jutila MA. Complex role of γδ T-cell-derived cytokines and growth factors in cancer. J Interferon Cytokine Res Off J Int Soc Interferon Cytokine Res (2012) 32(12):563–9. doi: 10.1089/jir.2012.0073 PMC3514002 23078623

[64] Brandes M Willimann K Moser B. Professional antigen-presentation function by human gammadelta T cells. Sci (New York NY). (2005) 309(5732):264–8. doi: 10.1126/science.1110267 15933162

[65] Landmeier S Altvater B Pscherer S Juergens H Varnholt L Hansmeier A. Activated human gammadelta T cells as stimulators of specific CD8+ T-cell responses to subdominant Epstein Barr virus epitopes: Potential for immunotherapy of cancer. J Immunother. (2009) 32(3):310–21. doi: 10.1097/CJI.0b013e31819b7c30 PMC3176339 19242369

[66] Maniar A Zhang X Lin W Gastman BR Pauza CD Strome SE. Human gammadelta T lymphocytes induce robust NK cell-mediated antitumor cytotoxicity through CD137 engagement. Blood. (2010) 116(10):1726–33. doi: 10.1182/blood-2009-07-234211 PMC3324253 20519625

[67] Liu Y Zhang C. The role of human γδ T cells in anti-tumor immunity and their potential for cancer immunotherapy. Cells. (2020) 9(5):111719. doi: 10.3390/cells9051206 PMC7290839 32413966

[68] Lee D Rosenthal CJ Penn NE Dunn ZS Zhou Y Yang L. Human &gamma;&delta; T cell subsets and their clinical applications for cancer immunotherapy. Cancers (Basel) [Internet]. (2022) 14(12):3005. doi: 10.3390/cancers14123005 35740670 PMC9221220

[69] Lo Presti E Pizzolato G Gulotta E Cocorullo G Gulotta G Dieli F. Current advances in γδ T cell-based tumor immunotherapy. Front Immunol (2017) 8:1401–. doi: 10.3389/fimmu.2017.01401 PMC5663908 29163482

[70] Rodriguez JB Falcone BN Szajnman SH. Approaches for designing new potent inhibitors of farnesyl pyrophosphate synthase. Expert Opin Drug Discovery. (2016) 11(3):307–20. doi: 10.1517/17460441.2016.1143814 26781029

[71] Lin JF Lin YC Lin YH Tsai TF Chou KY Chen HE. Zoledronic acid induces autophagic cell death in human prostate cancer cells. J urology. (2011) 185(4):1490–6. doi: 10.1016/j.juro.2010.11.045 21334668

[72] Dieli F Vermijlen D Fulfaro F Caccamo N Meraviglia S Cicero G. Targeting human {gamma}delta} T cells with zoledronate and interleukin-2 for immunotherapy of hormone-refractory prostate cancer. Cancer Res (2007) 67(15):7450–7. doi: 10.1158/0008-5472.CAN-07-0199 PMC3915341 17671215

[73] Fournié JJ Sicard H Poupot M Bezombes C Blanc A Romagné F. What lessons can be learned from γδ T cell-based cancer immunotherapy trials? Cell Mol Immunol (2013) 10(1):35–41. doi: 10.1038/cmi.2012.39 23241899 PMC4003170

[74] Siegers GM Lamb LS Jr. Cytotoxic and regulatory properties of circulating Vδ1+ γδ T cells: a new player on the cell therapy field? Mol Ther J Am Soc Gene Ther (2014) 22(8):1416–22. doi: 10.1038/mt.2014.104 PMC4435582 24895997

[75] Knowles LJ Malik M Nussebaumer O Brown A van Wetering S Koslowski M. Abstract CT525: GDX012U-001 a phase 1, open-label, dose escalation, and dose expansion study to assess the safety, tolerability, and preliminary antileukemic activity of GDX012 in patients with MRD positive AML. Cancer Res (2022) 82(12_Supplement):CT525–CT. doi: 10.1158/1538-7445.AM2022-CT525

[76] Corsale AM Di Simone M Dieli F Meraviglia S. Where could gammadelta T cells take us in the treatment of cancer? Expert Opin Biol Ther (2022) 23(1):1–5. doi: 10.1080/14712598.2022.2147424 36374510

[77] Ganesan R Chennupati V Ramachandran B Hansen MR Singh S Grewal IS. Selective recruitment of γδ T cells by a bispecific antibody for the treatment of acute myeloid leukemia. Leukemia. (2021) 35(8):2274–84. doi: 10.1038/s41375-021-01122-7 PMC8324575 33526858

[78] Yang R He Q Zhou H Gong C Wang X Song X. Vγ2 x PD-L1, a bispecific antibody targeting both the Vγ2 TCR and PD-L1, improves the anti-tumor response of Vγ2Vδ2 T cell. Front. Immunol. Vol. 13. (2022). 10.3389/fimmu.2022.923969 PMC9247338 35784353

[79] Broijl A NWCJvd D Bosch F Mateos M-V Rodríguez-Otero P Tucci A. Phase I dose escalation of LAVA-051, a novel bispecific gamma-delta T-cell engager (Gammabody), in relapsed/refractory hematological malignancies. Journal of Clinical Oncology Vol. 40. (2022). pp. 2577–.

[80] Makkouk A Yang XC Barca T Lucas A Turkoz M Wong JTS. Off-the-shelf Vδ1 gamma delta T cells engineered with glypican-3 (GPC-3)-specific chimeric antigen receptor (CAR) and soluble IL-15 display robust antitumor efficacy against hepatocellular carcinoma. J Immunother Cancer (2021) 9(12):e003441. doi: 10.1186/s12967-019-1897-0 34916256 PMC8679077

[81] Neelapu SS Stevens DA Hamadani M Frank MJ Holmes H Jacobovits A. A phase 1 study of ADI-001: Anti-CD20 CAR-engineered allogeneic gamma Delta1 (γδ) T cells in adults with b-cell malignancies. Blood (2022) 140(Supplement 1):4617–9. doi: 10.1182/blood-2022-157400

[82] Ganapathy T Radhakrishnan R Sakshi S Martin S. CAR γδ T cells for cancer immunotherapy. is the field more yellow than green? Cancer Immunology Immunotherapy (2022). doi: 10.1007/s00262-022-03260-y PMC10992831 35960333

[83] Izumi T Kondo M Takahashi T Fujieda N Kondo A Tamura N. Ex vivo characterization of γδ T-cell repertoire in patients after adoptive transfer of Vγ9Vδ2 T cells expressing the interleukin-2 receptor β-chain and the common γ-chain. Cytotherapy. (2013) 15(4):481–91. doi: 10.1016/j.jcyt.2012.12.004 23391461

[84] Bennouna J Levy V Sicard H Senellart H Audrain M Hiret S. Phase I study of bromohydrin pyrophosphate (BrHPP, IPH 1101), a Vgamma9Vdelta2 T lymphocyte agonist in patients with solid tumors. Cancer immunology immunotherapy CII. (2010) 59(10):1521–30. doi: 10.1007/s00262-010-0879-0 PMC11030967 20563721

[85] Noguchi A Kaneko T Kamigaki T Fujimoto K Ozawa M Saito M. Zoledronate-activated Vγ9γδ T cell-based immunotherapy is feasible and restores the impairment of γδ T cells in patients with solid tumors. Cytotherapy. (2011) 13(1):92–7. doi: 10.3109/14653249.2010.515581 20831354

[86] Nicol AJ Tokuyama H Mattarollo SR Hagi T Suzuki K Yokokawa K. Clinical evaluation of autologous gamma delta T cell-based immunotherapy for metastatic solid tumours. Br J Cancer. (2011) 105(6):778–86. doi: 10.1038/bjc.2011.293 PMC3171009 21847128

[87] Todaro M Orlando V Cicero G Caccamo N Meraviglia S Stassi G. Chemotherapy sensitizes colon cancer initiating cells to Vγ9Vδ2 T cell-mediated cytotoxicity. PloS One (2013) 8(6):e65145. doi: 10.3389/fimmu.2018.00800 23762301 PMC3675136

[88] Di Mascolo D Varesano S Benelli R Mollica H Salis A Zocchi MR. Nanoformulated zoledronic acid boosts the Vδ2 T cell immunotherapeutic potential in colorectal cancer. Cancers (Basel). (2019) 12(1):104. doi: 10.3390/cancers12010104 31906080 PMC7017311

[89] Ang WX Ng YY Xiao L Chen C Li Z Chi Z. Electroporation of NKG2D RNA CAR improves V9V2T cell responses against human solid tumor xenografts. Mol Ther - Oncolytics. (2020) 17:421–30. doi: 10.1016/j.omto.2020.04.013 PMC7240063 32462079

[90] Gadeta. . Available at: https://www.gadeta.nl/.

[91] Lu H Shi T Wang M Li X Gu Y Zhang X. B7-H3 inhibits the IFN-γ-dependent cytotoxicity of Vγ9Vδ2 T cells against colon cancer cells. Oncoimmunology. (2020) 9(1):1748991. doi: 10.1080/2162402X.2020.1748991 32363121 PMC7185217

[92] Li X Lu H Gu Y Zhang X Zhang G Shi T. Tim-3 suppresses the killing effect of Vγ9Vδ2 T cells on colon cancer cells by reducing perforin and granzyme b expression. Exp Cell Res (2020) 386(1):111719. doi: 10.1016/j.yexcr.2019.111719 31726050

[93] de Bruin RCG Veluchamy JP Lougheed SM Schneiders FL Lopez-Lastra S Lameris R. A bispecific nanobody approach to leverage the potent and widely applicable tumor cytolytic capacity of Vγ9Vδ2-T cells. Oncoimmunology. (2017) 7(1):e1375641–e. doi: 10.1080/2162402X.2017.1375641 PMC5739573 29296532

[94] Wu D Wu P Wu X Ye J Wang Z Zhao S. Ex vivo expanded human circulating Vδ1 γδT cells exhibit favorable therapeutic potential for colon cancer. Oncoimmunology. (2015) 4(3):e992749. doi: 10.4161/2162402X.2014.992749 25949914 PMC4404819

[95] Devaud C Rousseau B Netzer S Pitard V Paroissin C Khairallah C. Anti-metastatic potential of human Vδ1(+) γδ T cells in an orthotopic mouse xenograft model of colon carcinoma. Cancer immunology immunotherapy CII. (2013) 62(7):1199–210. doi: 10.1007/s00262-013-1402-1 PMC11028498 23619975

[96] Sebestyen Z Prinz I Déchanet-Merville J Silva-Santos B Kuball J. Translating gammadelta (γδ) T cells and their receptors into cancer cell therapies. Nat Rev Drug Discovery. (2020) 19(3):169–84. doi: 10.1038/s41573-019-0038-z 31492944

[97] van Diest E Hernández López P Meringa AD Vyborova A Karaiskaki F Heijhuurs S. Gamma delta TCR anti-CD3 bispecific molecules (GABs) as novel immunotherapeutic compounds. J ImmunoTherapy Cancer. (2021) 9(11):e003850. doi: 10.1136/jitc-2021-003850 PMC8611453 34815357

